# Reprogramming somatic cells to cells with neuronal characteristics by defined medium both *in vitro* and *in vivo*

**DOI:** 10.1186/s13619-015-0027-6

**Published:** 2015-12-30

**Authors:** Songwei He, Yiping Guo, Yixin Zhang, Yuan Li, Chengqian Feng, Xiang Li, Lilong Lin, Lin Guo, Haitao Wang, Chunhua Liu, Yi Zheng, Chuanming Luo, Qiang Liu, Fuhui Wang, Hao Sun, Lining Liang, Lingyu Li, Huanxing Su, Jiekai Chen, Duanqing Pei, Hui Zheng

**Affiliations:** 1 CAS Key Laboratory of Regenerative Biology, Guangdong Provincial Key Laboratory of Stem Cell and Regenerative Medicine, Guangzhou Institutes of Biomedicine and Health, Chinese Academy of Sciences, #190 Kaiyuan Avenue, Science City, Guangzhou, 510530 China; 2 State Key Laboratory of Quality Research in Chinese Medicine, Institute of Chinese Medical Sciences, University of Macau, Macau, China; 3 Anhui University, Hefei, 230601 China

**Keywords:** Neurons, Somatic cells, Astrocytes, Trans-differentiation, Defined medium

## Abstract

**Background:**

Currently, direct conversion from somatic cells to neurons requires virus-mediated delivery of at least one transcriptional factor or a combination of several small-molecule compounds. Delivery of transcriptional factors may affect genome stability, while small-molecule compounds may require more evaluations when applied *in vivo*. Thus, a defined medium with only conventional growth factors or additives for cell culture is desirable for inducing neuronal trans-differentiation.

**Results:**

Here, we report that a defined medium (5C) consisting of basic fibroblast growth factor (bFGF), N2 supplement, leukemia inhibitory factor, vitamin C (Vc), and β-mercaptoethanol (βMe) induces the direct conversion of somatic cells to cells with neuronal characteristics. Application of 5C medium converted mouse embryonic fibroblasts (MEFs) into TuJ+ neuronal-like cells, which were capable of survival after being transplanted into the mouse brain. The same 5C medium could convert primary rat astrocytes into neuronal-like cells with mature electrophysiology characteristics *in vitro* and facilitated the recovery of brain injury, possibly by inducing similar conversions, when infused into the mouse brain *in vivo*. Crucially, 5C medium could also induce neuronal characteristics in several human cell types.

**Conclusions:**

In summary, this 5C medium not only provides a means to derive cells with neuronal characteristics without viral transfection *in vitro* but might also be useful to produce neurons *in vivo* for neurodegenerative disease treatment.

**Electronic supplementary material:**

The online version of this article (doi:10.1186/s13619-015-0027-6) contains supplementary material, which is available to authorized users.

## Background

The successful generation of induced pluripotent stem cells (iPSCs) has greatly accelerated the research on stem cell and regenerative medicine [1], since iPSCs face fewer problems in ethics and immunological rejection when compared with embryonic stem cells (ESCs) [2, 3]. In addition, direct conversions from somatic cells to functional cells suggest that the intermediate pluripotent state could be avoided to decrease the possibility of tumorigenesis during cell transplantation [4, 5].

Engraftment of functional cells generated with stem cell-related technologies has the potential to revolutionize the treatment of neurodegenerative diseases [2]. Since the first report of the direct conversion from mouse embryonic fibroblasts (MEFs) to functional neurons [5], there have been many similar studies [6]. Although different aspects of these techniques have been improved, the direct conversion to neurons still requires virus-mediated delivery of at least one transcriptional factor [6–9], significantly limiting potential clinical applications. Genome integrations resulted from lentivirus- and retrovirus-mediated factor delivery that might reduce the stability of genome.

In addition, small-molecule compounds that regulate epigenetic modulation enzymes or signaling pathways are also frequently used during these direct conversions or the generation of induced pluripotent stem cells (iPSCs) [10–12]. Recently, trans-differentiation from somatic cells to neurons with different combinations of small-molecule compounds has been reported [13, 14]. Although the two reported recipes used different combinations of small-molecule compounds, they both used N2 supplement, B27 supplement, and basic fibroblast growth factor (bFGF) in their basal medium. Before the publication of these two recent reports, we had started developing a new defined medium with the following conventional growth factors or additives: N2 supplement, B27 supplement, bFGF, epidermal growth factor (EGF), leukemia inhibitory factor (LIF), vitamin C (Vc), and β-mercaptoethanol (βMe), for inducing direct conversions from somatic cells to neurons or cells with neuronal characteristics.

## Methods

### Medium for direct reprogramming and cell culture

Components of different mediums for direct reprogramming and cell culture are listed with their final concentrations and their catalog numbers in Additional file 1: Table S1. MEFs were derived from 13.5-day ICR mouse embryos. Normal MEFs in current studies were generated by removing the head and all internal organs, while MEFs sp- were generated by additionally removing the vertebral column (containing the spinal cord) and dorsal root ganglia [5]. Primary astrocytes were generated from rats within 1 day after birth [15]. All MEFs and astrocytes were cultured in FBS medium for additional two passages in non-coating plate after recovering from freezing in liquid nitrogen. MEFs and astrocytes that strongly attach to the plate were further enriched after removing cells with neurogenic potential and weak attachment by a 30-s 0.25 % trypsin treatment. Mouse macrophages, human foreskin fibroblasts (HFFs), and mesenchymal stem cells from human bone marrow (BM-hMSCs) were prepared as reported previously [16, 17]. Human cells were collected with the informed consent, and the usages have been approved by related ethics committees in each institute or university. These cells were maintained in FBS medium before conversion.

MEFs were plated at 50,000 per well after coating the 6-well plate with Matrigel coating for 0.5 h. 5C medium was used for 16 days after replating. Half medium was replaced with fresh medium every 2 days. When indicated, neuron culture (NC) medium was used for additional culturing from day 16 to day 24 with half medium changed every 2 days. Conversions of other cells followed the same protocol, except as annotated specifically.

### FACS

Paraformaldehyde-fixed cells or 10-μM cryostat sections from mice perfused with 4 % paraformaldehyde were washed with PBS twice and blocked with PBS containing 10 % goat serum, 1 % BSA, and 0.3 % TritionX-100. Antibodies were diluted with PBS containing 1 % goat serum, 1 % BSA, and 0.3 % Trition X-100 and incubated with the samples for 12 and 2 h, respectively, at room temperature. Three times washing with PBS followed each antibody incubation. Immunofluorescence was detected with Zeiss LSM710, and immunofluorescence and fluorescence-activated cell sorting (FACS) assay was performed with BD Accuri C6 flow cytometer. Antibodies were listed in Additional file 1: Table S2.

### Animal studies

Our studies followed the guidelines for the Care and Use of Laboratory Animals of the National Institutes of Health. The protocols were approved by the Committee on the Ethics of Animal Experiments of Guangzhou Institutes of Biomedicine and Health. All efforts were made to minimize animal discomfort.

About 50,000 cells collected on day 10 of the conversion were engrafted into the lateral ventricle (0.7 mm posterior to the bregma, lateral ±1.2 mm, and 2.4 mm to the skull) of 2-month NSI mice (NOD/SCID/IL2rg−/−, with minimal B, T, and NK cells) after being labeled with EGFP via lentivirus system. The survival of the cells was examined 4 weeks later. 5C medium or saline were infused into mouse brain (2.0 mm posterior to the bregma, lateral 1.2 mm, and 3.2 mm to the skull) with osmatic minipump (0.5 μl/h, 14 days), and brain slides were analyzed at indicated time points.

### RNA-seq

RNA was extracted from cells using TRIzol reagent (Invitrogen). Illumina mRNA-seq libraries were prepared for each RNA sample by using the TruSeq RNA Sample Preparation Kit v2 (Illumina) before sequencing on an Illumina MiSeq instrument with the MiSeq Reagent Kit (Illumina).

Obtained RNA-Seq reads were processed by RSEM (RNA-Seq by Expectation-Maximization) to estimate transcript abundances. Reads were aligned to a synthetic transcriptome, and the number of reads associated with a given transcript was used to estimate that transcript’s abundance in TPM (transcripts per million).

### Electrophysiological analysis

Whole-cell patch-clamp recording techniques were used to study the physiological properties of TuJ+ cells in culture with borosilicate glass pipettes (resistance 5–10 MΩ) using an Axopatch 200B amplifier (Axon Instruments for Molecular Devices). Both the spontaneous postsynaptic current and current response to exogenous focal application of glutamate and GABA were recorded. Pressure ejection was used to puff 1 mM glutamate (10 p.s.i., 100 ms) and 1 mM GABA (10 p.s.i., 100 ms), and the holding voltage was −70 mV. The patch pipette internal solution contained (in mM) 136.5 K-gluconate, 0.2 EGTA, 10 HEPES, 9 NaCl, 17.5 KCl, 4 Mg-ATP, and 0.3 Na-GTP, adjusted with KOH to pH 7.2, 285 osmol/l. The external solution contained (in mM) 120 NaCl, 1.2 KH2PO4, 1.9 KCl, 26 NaHCO_3_, 2.2 CaCl_2_, 1.4 MgSO_4_, 10 d-glucose, and 7.5 HEPES (pH with NaOH to 7.3). The bath solution was equilibrated with 95 % O_2_ and 5 % CO_2_ before use. In current clamp mode, membrane potentials were generally maintained at about −60 mV by injecting a small bias current. Signals were sampled at 10 kHz using a Digidata1440A analog-to-digital converter and acquired and stored on a computer hard drive using pClamp10 software. Data were analyzed using pClamp10 (Clampfit).

### Accession numbers

Current RNA-seq data were deposited in the Gene Expression Omnibus under accession number: GSE68902. Please use the following link to get access to the RNA-seq data (http://www.ncbi.nlm.nih.gov/geo/query/acc.cgi?token=qzclyiiuhrolbux&acc=GSE68902).

### Statistical methods

Experiments were repeated at least three times (*n* ≥ 3). Data were analyzed and compared by two-tailed *t* test or one-way ANOVA with Dunnett’s test as a post hoc. Error bars represent standard deviations (or standard error of the mean, if specifically mentioned) and “*n*” represents the number of independent experiments. “*”, “**”, and “***” represent significant differences (*p* < 0.05), (*p* < 0.01), and (*p* < 0.001) from indicated control groups, respectively.

## Results

### 5C medium converts MEFs into cells with neuronal characteristics

**Fig. 1:**
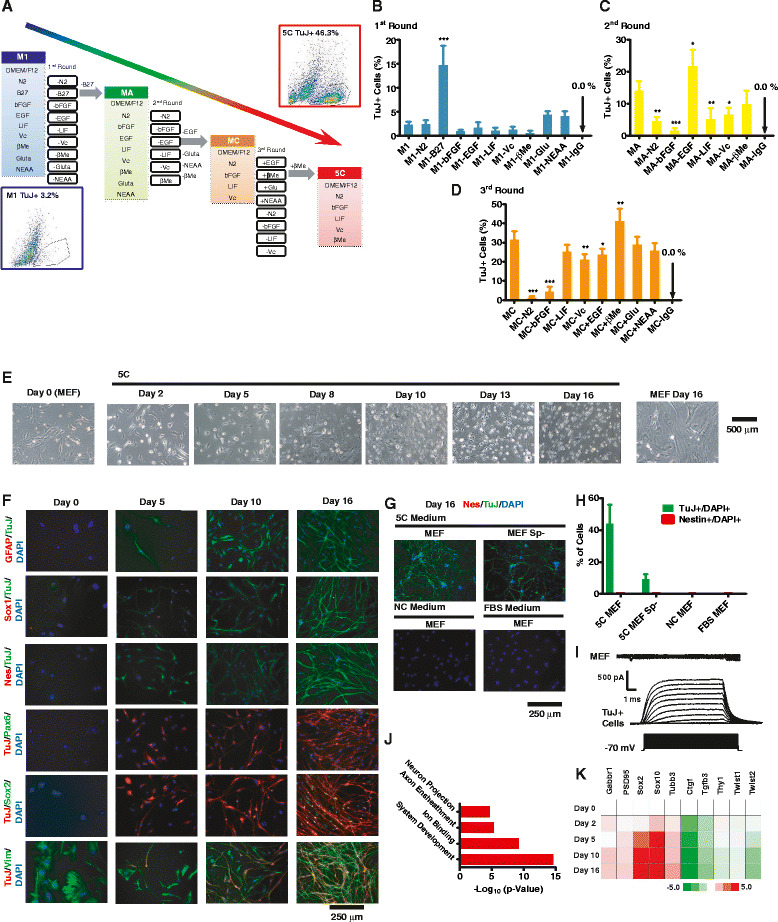
Development of 5C medium that directly converts MEFs to cells with neuronal characteristics. **a** Schematic illustration of the steps taken to develop the 5C medium (conversion efficiency as high as 46.3 %) from MC, MA, and the original M1 medium (efficiency around 3.2 %) during the first three rounds of factor deduction. **b**–**d** Percentages of TuJ+ cells determined by FACS during four rounds of factor reduction. The components of all medium were listed in Additional file 1: Table S1. Antibody against IgG was used as a negative control during FACS analysis. **e** Morphologies of cells on days 0, 2, 5, 8, 10, 13, and 16 during reprogramming were provided as phase-contrast images. **f** GFAP, Sox1, Nestin (Nes), Pax6, Sox2, or Vim were stained together with TuJ and DAPI on days 0, 5, 10, and 16 during reprogramming. **g**–**h** MEFs generated without the spinal cord (sp-), normal MEFs were cultured with 5C medium for 16 days and subjected for TuJ and Nestin staining. Another two groups of MEFs were cultured with NC medium and FBS medium, respectively. Nestin, TuJ, and DAPI were stained on day 16 (**g**). Percentage of Nestin+ and TuJ+ cells was summarized in **h**. **i** Representative recordings of voltage-gated ion channels from a TuJ+ cells. **j**–**k** RNA-seq were performed on days 0, 2, 5, 10, and 16 during 5C-induced trans-differentiation. Genes with significant up-regulation (over twofold) were used for Gene Ontology analysis. The top four enriched clusters were listed in **j**. The expression of five neuron markers and five fibroblast markers from RNA-seq was listed in **k**

In preliminary studies, M1 medium (DMEM/F12 supplemented with N2, B27, bFGF, EGF, LIF, Vc, βMe, GlutaMax (Glu), and non-essential amino acids (NEAA)) could induce significant morphological changes of MEFs and generate TuJ+ cells at a low percentage (3.2 %) on day 16 (Fig. 1a). We next sought to optimize the generation of TuJ+ cells and so to develop an optimal medium by testing dropouts of each medium component (Fig. 1a, Additional file 1: Figure S1 and Table S1).

M1 medium was used in the first round of factor deduction, and only the removal of B27 increased the percentage of TuJ+ cells on day 16 (Fig. 1b). Thus, B27 was removed from M1 medium to form a new MA medium. MA medium was used in the second round of factor deduction; the removal of EGF led to the generation of 22 % TuJ+ cells (Fig. 1c). In addition, Glu, NEAA, and βMe were not essential (Fig. 1b,c). Thus, MC medium was prepared with N2, bFGF, LIF, and Vc and tested in the third round of factor deduction. Re-addition of βMe to MC medium was the optimal recipe and could result in approximately 40 % of cells becoming TuJ+ on day 16 (Fig. 1d).

5C medium induced the majority of MEFs to undergo morphological changes including the shrinking of cell and nuclear sizes, the formation and elongation of cell surface protrusions at multiple points, and the adoption of typical neuronal morphology (Fig. 1e). TuJ+ cells were observed as early as day 5 though with a morphology closer to fibroblasts than typical neurons. The percentage of TuJ+ cells increased, and their morphologies changed towards neurons during the following conversion. No GFAP+, Nestin+, or Pax6+ cells were observed during the whole progress (Fig. 1f). About 1 % Sox2+ and less than 0.1 % Sox1+ cells were observed on day 16. The fibroblast marker, vimentin (Vim), was also stained during the conversion. As indicated in Fig. 1f, cells positive for both Vim and TuJ could be observed since day 5, and a large portion of TuJ+ cells were also positive for Vim on day 16, suggesting the gradual conversion from MEFs to cells with neuronal characteristics.

TuJ+ cells were also observed though with a lower efficiency (Fig. 1g, h), when MEFs (sp-) were prepared by removing the head, vertebral column (containing the spinal cord), dorsal root ganglia, and all internal organ spinal cords as reported previously and subjected for reprogramming [5]. Although it was still difficult to exclude all the possible involvement of cells with neurogenic potential, it was reasonable to suggest an exclusion of majority. However, NC medium, a medium that can be used for neuron progenitor/stem cell (NPC) differentiation and neuron culture [15, 18, 19], and FBS medium (Additional file 1: Table S1) did not induce similar morphological changes or TuJ+ cells from MEFs (Fig. 1g, h). Therefore, the MEFs used in current studies do not contain NPCs, neurons, or cells that could easily differentiate to neurons and 5C medium can specifically induce the conversion of MEFs to TuJ+ cells.

Electrophysiology studies of the resulting cells suggested that these TuJ+ cells have some characteristics of neurons, like voltage-gated potassium currents (Fig. 1i), but the cells failed to demonstrate sodium current or spontaneous postsynaptic currents (data not shown). Thus, the 5C medium only convert MEFs to a state close to neurons. To get more information on this conversion, RNA-seq analysis was performed on days 2, 5, 10, and 14 during the conversion (GSE68902). RNA-seq analysis during the process also suggested a conversion from MEFs to cells with neuronal characteristics (Additional file 2: Table S3). Gene Ontology analysis suggested that 5C medium directed MEFs towards the acquisition of neural characteristics (Fig. 1j). Trans-differentiation was also suggested by the up-regulation of neuron markers like TuJ, GABA receptor, and PSD95 and the down-regulation of fibroblast markers like Thy1 and Twists (Fig. 1k).

However, when comparing the expression profiles of cells on day 16 to mature neurons, the generated cells were still closer to MEFs, which was consistent with the significant Vim staining on day 16 in Fig. 1g. In addition, the observed up-regulation of Sox2 in RNA-seq dataset was not fully consistent with the small percentage of Sox2+ cells on day 16. Since current antibodies against Sox2 work well in NPCs and in previous report [20], the inconsistency might be due to increased P300 expression during conversion (Additional file 2: Table S3), which could induce Sox2 acetylation and degradation [21].

### TuJ+ cells survive after transplantation

**Fig. 2:**
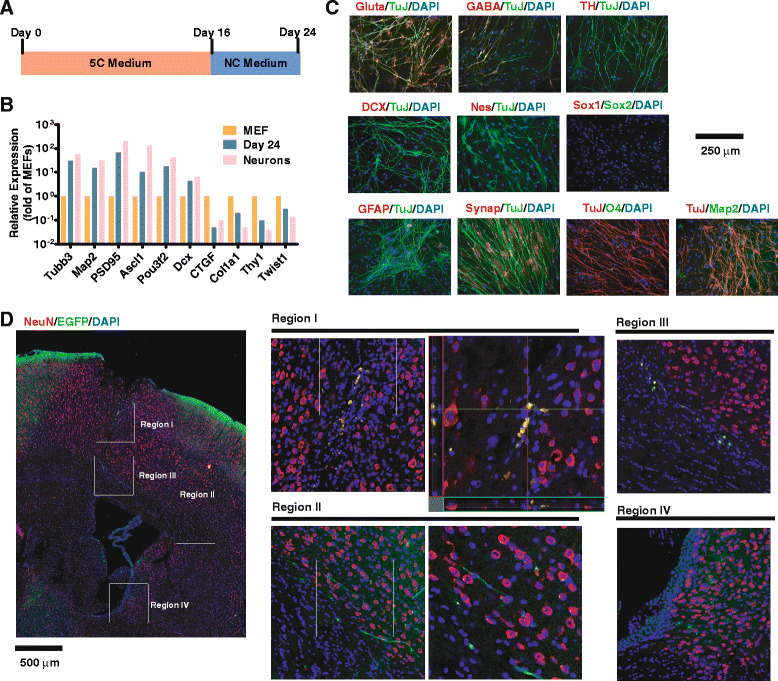
TuJ+ cells are further converted towards neuron-like cells. **a** Schematic illustration of 5C and NC medium culturing for MEFs. **b** Gene expression on day 24 were determined by qPCR and compared with those in MEFs. **c** MEFs were cultured with 5C medium for 16 days and with NC medium for an additional 8 days. Glutamate, GABA, TH, GFAP, synapsin, O4, Map2, DCX, Nestin, Sox1, Sox2, and TuJ+ were stained on day 24 as indicated. **d** About 50,000 cells on day 12 were labeled with EGFP and engrafted into the mouse brain. Their survival and co-staining with NeuN were determined 21 days after engraftment. The regions I–IV were marked in the first picture and enlarged by four times. Regions with interest in regions I–II were also marked and enlarged by another two times. Scale bars were provided for the largest image

In addition, since only a small portion of TuJ+ cells were stained positive for Map2 on day 16 during reprogramming, NC medium was used to culture the cells for an additional 8 days for maturation (Fig. 2a). The expression of neuronal markers like TuJ, Map2, and Ascl1 was significantly increased, while fibroblast markers were decreased (Fig. 2b). In addition, NC medium further resulted in cells positive for Map2, GABA, glutamate, and synapsin I (Fig. 2c), all markers of enhanced commitment to a neuronal fate. However, NC medium still did not generate cells with the full electrophysiological characteristics of mature neurons. In addition, consistent with Fig. 1g, a few cells (less than 0.5 %) were positive for Sox2 on day 24, which was less than the percentage of Sox2+ cells on day 16 and suggested that NC medium did not induce significant number of Sox2+ cells or NPC-like cells.

To determine whether these TuJ+ cells formed functional neurons within an appropriate *in vivo* niche, about 50,000 cells collected on day 10 of the conversion were engrafted into the lateral ventricle (0.7 mm posterior to the bregma, lateral ±1.2 mm, and 2.4 mm to the skull) of 2-month NSI mice (NOD/SCID/IL2rg−/−, with minimal B, T, and NK cells) after being labeled with EGFP via lentivirus system. Surviving EGFP+ cells were observed along the injection route 3 weeks after engraftment. About 30 % of surviving EGFP+ cells were also stained positively with the mature neuron marker, NeuN (Fig. 2d), a marker we had not observed in culture, suggesting that our culture-derived TuJ+ cells could survive and showed enhanced maturation inside the mouse brain. The different morphologies of survived cells in four regions listed in Fig. 2d suggested the different levels of maturations of these cells, possibly due to the differences in the stages during conversion process or microenvironment.

### 5C medium converts astrocytes to mature neurons

**Fig. 3:**
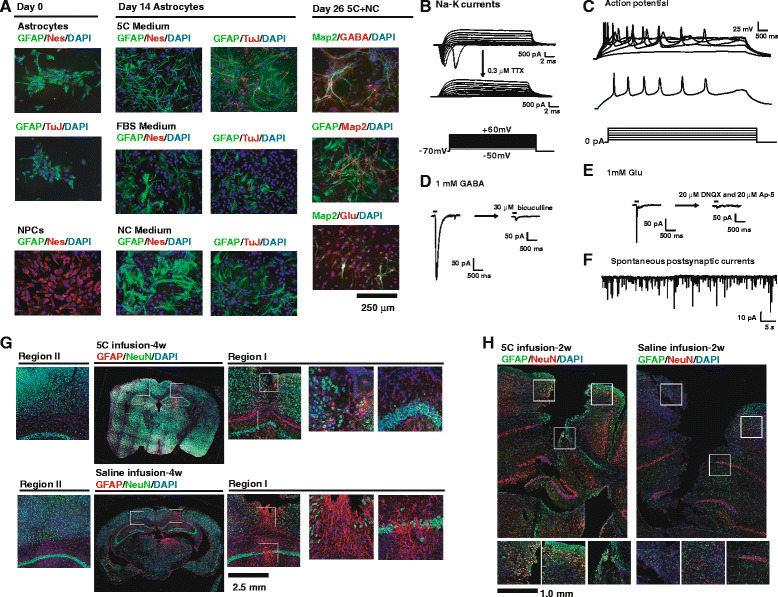
5C medium converts astrocytes to functional neurons. **a** Rat astrocytes and mouse NPCs were stained for GFAP, TuJ, and Nestin on day 0. Rat astrocytes were then cultured with 5C, FBS, and NC medium for 14 days and were stained for Nestin and TuJ. NC medium was used for additional 12 days before further characterization with antibodies against GABA, glutamate, and Map2. **b**–**f** Rat astrocyte-converted neurons are functional *in vitro* at day 26. Representative recordings of voltage-gated ion channels from an astrocyte-converted neuron. Both an outward current and an inward current were observed, and the inward currents were blocked by tetrodotoxin (*TTX*). **b** Astrocyte-converted neurons showed neuronal typical trains of action potentials in response to intracellular step current injections of 10, 20, 30, 40, 50, and 60 pA. **c** Astrocyte-converted neurons showed bicuculline-sensitive inhibitory postsynaptic currents in response to GABA puffs (**d**), DNQX-AP5-sensitive excitatory postsynaptic currents in response to glutamate puffs (**e**), and spontaneous postsynaptic currents (PSCs) (**f**). **g**–**h** 5C medium and saline were infused into the mice brain for 14 days. Brain slides were analyzed on day 28 (**g**) and on day 14 (**h**) with antibodies against GFAP and NeuN. In **g**, regions I–II were marked in the largest pictures and enlarged by six times. The region with interest in region I of two groups was also marked and enlarged by another four times. In **h**, the region with interest was marked and enlarged by two times. Scale bars were provided for the largest image in each panel

Primary rat astrocytes were isolated and further cultured on uncoated plates with FBS medium for two passages to remove any contaminations of NPCs and neurons. After culturing these rat astrocytes with 5C medium for 14 days, NC medium was used for additional 12 days for maturation. As indicated in Fig. 3a, 5C medium induced the direct conversion of astrocytes to TuJ+ cells within 14 days, while no Nestin+ cells were identified. In addition, neither FBS nor NC medium induced similar reprogramming of astrocytes. If NC medium was used to culture the cells from day 15 to day 26, significant percentages of cells positive for Map2, GABA, and glutamate were identified (Fig. 3a). Some of these Map2+ cells have spontaneous postsynaptic currents and other electrophysiology characteristics of mature neurons (Fig. 3b–f) indicating the conversion of astrocytes to functional neurons. The astrocyte-converted cells were closer to mature neurons than those from MEFs, possibly because of the neurogenic factors secreted by the remained astrocytes and the higher neuronal background of astrocytes.

As a combination of conventional growth factors and small-molecule compounds, 5C medium can be applied *in vivo* easier and safer than other methods. Thus, 5C medium or saline were infused into the mouse brain (2.0 mm posterior to the bregma, lateral 1.2 mm, and 3.2 mm to the skull) with osmatic minipump (0.5 μl/h, 14 days), and brain slides were analyzed after additional 14 days. We did not infuse the medium or saline in the lateral ventricle where the generated cells were transplanted into in Fig. 2d because the large volume of the lateral ventricle was easier for the infused medium to diffuse and was quicker to reduce the local concentration of components in the medium.

As indicated in Fig. 3g, long-term infusion significantly damaged the mouse brain. NeuN+ cells decreased while GFAP+ cells increased significantly around the wound when comparing the infusion side (region I) with the non-surgical side (region II). However, in mice infused with 5C medium, the damage to the brain was partially recovered as indicated by the increased NeuN+ cells and decreased GFAP+ cells, when compared to mice brains infused with saline.

These increased NeuN+ cells around injury sites after 5C infusion could be explained by the potential abilities of 5C medium to recruit NPCs to the wound and relieve the damage after differentiation or to protect NeuN+ cells with additional nutrition. To exclude these two possibilities, mice were analyzed just after finishing the infusion on day 14. There was a significant lesion on day 14 slides because the infusion pumps were removed just on day 14, which left no time for the wound to recover. As indicated in Fig. 3h, a significant number of NeuN+/GFAP+ cells were observed 2 weeks after 5C infusion, while few was observed in saline-infused mice. Since NeuN+/GFAP+ cells were not observed during normal differentiation of NPCs or in NeuN+ cells [22], the increased number of NeuN+ cells around wounds did not result from NPC differentiation or NeuN+ cell protection. Therefore, although additional evidences are required to further confirm the connection between these NeuN+/GFAP+ cells and astrocyte-to-neuron conversion, we can suggest that 5C medium increases NeuN+ cells around wounds possibly via the conversion from astrocytes to NeuN+ cells rather than NPC differentiation.

### 5C is also applicable for several human cells

We next explored the utility of 5C to convert other mouse somatic cells and also human somatic cells to TuJ+ cells, 5C medium induced TuJ+ cells from mouse macrophages, and BM-hMSCs (Additional file 1: Figure S2A). HFFs could not be converted to TuJ+ cells by 5C medium alone, but the addition of VPA, CHIR99021, A8-301, and forskolin (only after day 6) (Additional file 1: Table S1; based on previous reports [10, 11, 18]) could now lead to the conversion of HFFs to TuJ+ cells at a relatively high efficiency (Additional file 1: Figure S2B). However, since the conversion in human system (HFFs) seemed to be difficult than in mouse system (MEFs), additional investigation or experiments should be performed to further confirm and demonstrate the conversion in human system.

## Discussion

The current studies reported a two-phase method with defined medium for trans-differentiation, which can induce MEFs to cells with neuronal characteristics or induce astrocytes to cells with mature electrophysiology characteristics. In the 16-day phase I, the current 5C medium induced MEFs to TuJ+ cells. The RNA-seq suggested that although these TuJ+ cells expressed some neuronal markers, they were still closer to MEFs than to mature neurons. This observation might be due to the majority (60 %) of cells on day16 being still MEFs and negative for TuJ staining. Since these TuJ+ cells on day 16 only had voltage-gated potassium currents and were also stained positive for Vim, we classified these cells as cells with neuronal characteristics or neuron-like cells. With the help of NC medium in phase II, cells generated form MEFs could obtain higher expression of neuronal markers, and cells from astrocytes could have electrophysiology characteristics of mature neurons. In addition, we failed to generate cells with more neuronal characteristics when applying the maturation medium used in two recent reports [13, 14] or using additional neuronal growth factors like GDNF and so on in phase II (data not shown). However, with additional investigation including other growth factors and passaging the cells on glia monolayers might still be possible to promote enhanced neuronal characteristics of generated cells.

Heads and internal organs were already removed during the preparation of normal MEFs, cells but the spinal cord could still be a source for cells with neurogenic potential. However, removing the spinal cord to generate MEFs (sp-) only reduced the TuJ+ cells from about 40 % to about 15 %, which suggested the ability of 5C to convert cells without neurogenic potential. In addition, the difference in TuJ+ cells generated from normal MEFs and MEFs (sp-) suggested that the cells with neurogenic potential in the spinal cord were easier to be induced to TuJ+ cells by 5C medium. The cells with neurogenic potential in MEFs were definitely not NPCs or neurons because of the negative immune-staining for related markers and the two-passage culturing in FBS medium and non-coating plate.

Cells with neurogenic potential should be easier for conversion, which was reasonable. The question was what kind or what level of neurogenic potential was necessary or sufficient. However, it is hard to define or quantify the neurogenic potential and potential for other lineages. Since normal MEFs or MEFs (sp-) were prepared around E13.5, they should inherit some epigenetic traces from ESCs and have certain potential for multiple lineages. Astrocytes were in the same lineage as neurons, while BM-hMSCs also have neurogenic potential as suggested previously [23]. The macrophages and HFFs had little neurogenic potential, but only macrophages could be converted by 5C medium. Thus, the level of neurogenic potential might not be a critical factor for current conversion and additional investigation should be performed for further understanding.

The current 5C medium provides a new potential method to treat related neuronal diseases. Since the current medium was applicable in both mouse brain *in vivo* and rat astrocytes system *in vitro*, it is reasonable to suggest its functions in human astrocytes and human brain system though no preliminary data was provided here. Additional investigation with human cells was required before further discussion on clinical application.

If LIF, Vc, and βme were removed from 5C medium, a 2C medium could be generated. Such 2C medium could induce TuJ+ cells at a lower but acceptable percentage (about 25 %, bar 4–5 in Fig. 1d and data not shown). Since our defined medium could use fewer components than previous reports, it should be closer to clinical application. Furthermore, if insulin, transferrin, and other components in N2 were capable to replace N2 supplement during the trans-differentiation, it should be possible to reduce the factors in medium to a level even lower and to facilitate the possible clinical application.

## Conclusions

The current defined medium was capable to induce direct conversion of MEFs to cells with neuronal characteristics. In addition, this medium could also induce similar conversions on primary rat astrocytes *in vitro* and in mouse brain when infused *in vivo*.

## Additional files

Additional file 1: **Figures S1–S2 and Tables S1–S2.** **Figure S1.** Percentage of TuJ+ cells generated on day 16 with different mediums MEFs were cultured with indicted mediums for 16 days. Percentage of TuJ+ cells was determined with FACS analysis. *y*-axis and *x*-axis represented SSC and Alexa 488 reads. The current figure was used to support Fig. 1b–d. **Figure S2.** 5C medium converts other cells to TuJ+ cells. Macrophages, BM-hMSCs (A), and HFFs (B) were cultured with 5C or 5C plus (HFFs, plus A8-301, CHR99021, VPA and Forskolin, **Table S1**) mediums for 16 days. TuJ + cells were presented. **Table S1.** List of growth factors and chemicals used in current studies. The purchase information and final concentrations of growth factors/small-molecule compounds were provided below. Recipes of different medium used in current studies were also listed by providing “Y” in designed boxes of their components. The growth factors/small-molecule compounds were included in related medium for all the time, while only forskolin was only used after day 6 during culturing. The basal medium was made from 1:1 neurobasal and F12 medium. NC medium was used before mixing one half fresh medium with another half that had been incubated with astrocytes for 24 h. **Table S2.** List of antibodies used in the current study. (PDF 402 kb)

Additional file 2: Table S3. Normalized RNA-seq results during conversion from MEFs to neuron-like cells. (XLSX 1144 kb)

## Abbreviations

bFGF: basic fibroblast growth factor
βMe: β-mercaptoethanol
BM-hMSCs: mesenchymal stem cells from human bone marrow
EGF: epidermal growth factor
ESCs: embryonic stem cells
Glu: GlutaMax
HFFs: human foreskin fibroblasts
iPSCs: induced pluripotent stem cells
LIF: leukemia inhibitory factor
MEFs: mouse embryonic fibroblasts
NEAA: non-essential amino acids
NC: neuron culture
Vc: vitamin C

## References

[1] Takahashi K, Yamanaka S (2006). Induction of pluripotent stem cells from mouse embryonic and adult fibroblast cultures by defined factors. Cell.

[2] Nishikawa S, Goldstein RA, Nierras CR (2008). The promise of human induced pluripotent stem cells for research and therapy. Nat Rev Mol Cell Biol.

[3] Li M, Chen M, Han W, Fu X (2010). How far are induced pluripotent stem cells from the clinic?. Ageing Res Rev.

[4] Ieda M, Fu JD, Delgado-Olguin P, Vedantham V, Hayashi Y, Bruneau BG (2010). Direct reprogramming of fibroblasts into functional cardiomyocytes by defined factors. Cell.

[5] Vierbuchen T, Ostermeier A, Pang ZP, Kokubu Y, Sudhof TC, Wernig M (2010). Direct conversion of fibroblasts to functional neurons by defined factors. Nature.

[6] Xu J, Du Y, Deng H (2015). Direct lineage reprogramming: strategies, mechanisms, and applications. Cell Stem Cell.

[7] Torper O, Pfisterer U, Wolf DA, Pereira M, Lau S, Jakobsson J (2013). Generation of induced neurons via direct conversion *in vivo*. Proc Natl Acad Sci U S A.

[8] Xue Y, Ouyang K, Huang J, Zhou Y, Ouyang H, Li H (2013). Direct conversion of fibroblasts to neurons by reprogramming PTB-regulated microRNA circuits. Cell.

[9] Guo Z, Zhang L, Wu Z, Chen Y, Wang F, Chen G (2014). *In Vivo* direct reprogramming of reactive glial cells into functional neurons after brain injury and in an Alzheimer’s disease model. Cell Stem Cell.

[10] Cheng L, Hu W, Qiu B, Zhao J, Yu Y, Guan W, et al. Generation of neural progenitor cells by chemical cocktails and hypoxia. Cell Res. 2014. 10.1038/cr.2014.32 PMC4042166 24638034

[11] Hou P, Li Y, Zhang X, Liu C, Guan J, Li H (2013). Pluripotent stem cells induced from mouse somatic cells by small-molecule compounds. Science.

[12] Wang L, Wang L, Huang W, Su H, Xue Y, Su Z (2012). Generation of integration-free neural progenitor cells from cells in human urine. Nat Methods.

[13] Li X, Zuo X, Jing J, Ma Y, Wang J, Liu D (2015). Small-molecule-driven direct reprogramming of mouse fibroblasts into functional neurons. Cell Stem Cell.

[14] Hu W, Qiu B, Guan W, Wang Q, Wang M, Li W (2015). Direct conversion of normal and Alzheimer’s disease human fibroblasts into neuronal cells by small molecules. Cell Stem Cell.

[15] Kaech S, Banker G (2006). Culturing hippocampal neurons. Nat Protoc.

[16] Weng JY, Du X, Geng SX, Peng YW, Wang Z, Lu ZS (2010). Mesenchymal stem cell as salvage treatment for refractory chronic GVHD. Bone Marrow Transplant.

[17] Panula S, Reijo Pera RA (2008). Preparation of human foreskin fibroblasts for human embryonic stem cell culture. CSH Protocols.

[18] Wang L, Wang L, Huang W, Su H, Xue Y, Su Z (2013). Generation of integration-free neural progenitor cells from cells in human urine. Nat Methods.

[19] Su H, Wang L, Huang W, Qin D, Cai J, Yao X (2013). Immediate expression of Cdh2 is essential for efficient neural differentiation of mouse induced pluripotent stem cells. Stem Cell Res.

[20] Suh H, Consiglio A, Ray J, Sawai T, D’Amour KA, Gage FH (2007). *In vivo* fate analysis reveals the multipotent and self-renewal capacities of Sox2+ neural stem cells in the adult hippocampus. Cell Stem Cell.

[21] Baltus GA, Kowalski MP, Zhai H, Tutter AV, Quinn D, Wall D (2009). Acetylation of sox2 induces its nuclear export in embryonic stem cells. Stem Cells.

[22] von Bohlen Und Halbach O (2007). Immunohistological markers for staging neurogenesis in adult hippocampus. Cell Tissue Res.

[23] Ng TK, Fortino VR, Pelaez D, Cheung HS (2014). Progress of mesenchymal stem cell therapy for neural and retinal diseases. World J Stem Cells.

