# Supplementary material for: Reprogramming somatic cells to cells with neuronal characteristics by defined medium both in vitro and in vivo
Source: Cell Regen. 2015 Dec 30;4:12. doi: 10.1186/s13619-015-0027-6 (PMC4696146; doi:10.1186/s13619-015-0027-6)
Supplement: Supplementary file 1 — Figures S1–S2 and Tables S1–S2. Figure S1. Percentage of TuJ+ cells generated on day 16 with different mediums MEFs were cultured with indicted mediums for 16 days. Percentage of TuJ+ cells was determined with FACS analysis. y-axis and x-axis represented SSC and Alexa 488 reads. The current figure was used to support Fig. 1b–d. Figure S2. 5C medium converts other cells to TuJ+ cells. Macrophages, BM-hMSCs (A), and HFFs (B) were cultured with 5C or 5C plus (HFFs, plus A8-301, CHR99021, VPA and Forskolin, Table S1) mediums for 16 days. TuJ + cells were presented. Table S1. List of growth factors and chemicals used in current studies. The purchase information and final concentrations of growth factors/small-molecule compounds were provided below. Recipes of different medium used in current studies were also listed by providing “Y” in designed boxes of their components. The growth factors/small-molecule compounds were included in related medium for all the time, while only forskolin was only used after day 6 during culturing. The basal medium was made from 1:1 neurobasal and F12 medium. NC medium was used before mixing one half fresh medium with another half that had been incubated with astrocytes for 24 h. Table S2. List of antibodies used in the current study. (PDF 402 kb) [file 13619_2015_27_MOESM1_ESM.pdf]

Supplementary Information

Supplementary Figures

Figure S1

Percentages of TuJ+ cells generated on Day 16 with different mediums

MEFs were cultured with indicated mediums for 16 days. Percentage of TuJ+ cells were determined with FACS analysis. Y-axis and X-axis represented SSC and Alexa 488 reads. The current Figure was used to support Figure 1B-D.

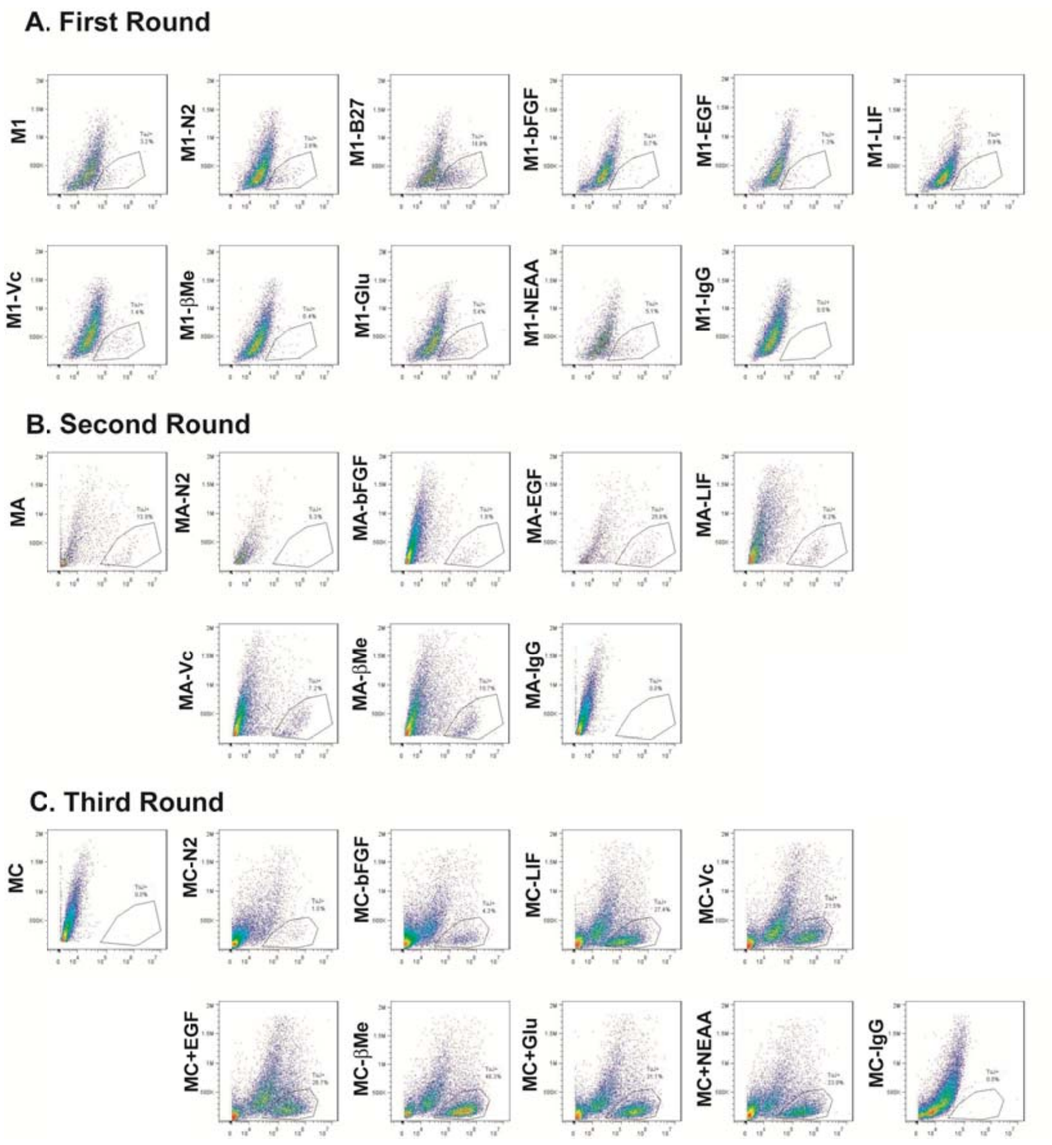

Figure S2

5C medium converts other cells to TuJ+ cells

Macrophages, BM-hMSCs (A), and HFFs (B)were cultured with 5C or 5C plus (HFFs, plus A8-301, CHIR99021, VPA and Forskolin, Table S1) medium for 16 days. TuJ+ cells were presented.

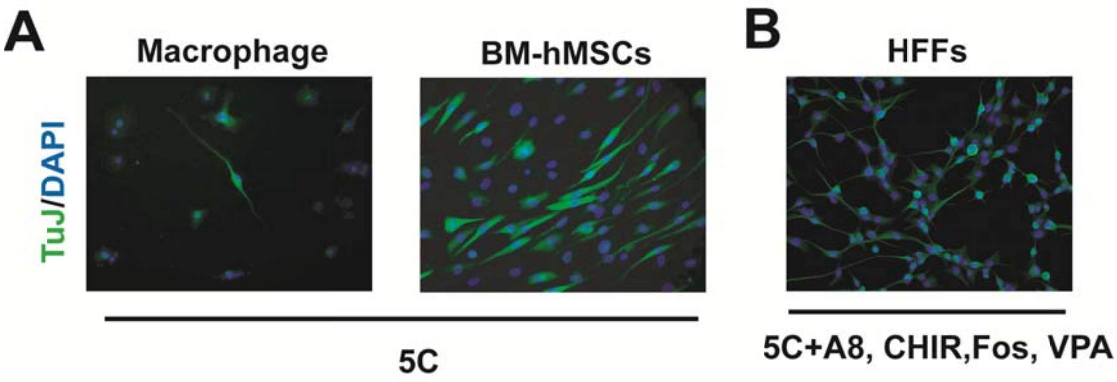

Supplementary Tables

Table S1

List of growth factors and chemicals used in current studies

The purchase information and final concentrations of growth factors/small molecule compounds were provided below. Recipes of different medium used in current studies were also list by providing “Y” in designed boxes of their components. The growth factors/small molecule compounds were included in related medium for all the time, while only forskolin was only used after Day 6 during culturing. The basal medium was made from 1:1 neurobasal and F12 medium. NC medium was used before mixed one half fresh medium with another half that had been incubated with astrocytes for 24 hours.

	Company	Cat. No.	Final Con.	FBS	M1	MA	MC	5C	5C(HFFs)	NC
**DMEM/F12**	Gibco	11330-032	Basal	Y	Y	Y	Y	Y	Y	
**F12**	Gibco	21127-022	Basal							Y 50%
**Neurobasal**	Gibco	21103-049	Basal							Y 50%
**FBS**	Excell	FSP500	10%	Y						
**GlutaMAX**	Gibco	35050-061	0.1%	Y	Y	Y				
**NEAA**	Gibco	11140-050	0.1%	Y	Y	Y				
**N2**	Gibco	17502-048	1%		Y	Y	Y	Y	Y	Y
**B27**	Gibco	17504-044	2%		Y					Y
**bFGF**	Self Made	Ref.[1]	20 ng/ml		Y	Y	Y	Y	Y	

**EGF**	PeproTech	AF-100-15	10 ng/ml		Y	Y				
**LIF**	Self Made	Ref. [2]	0.01 μg/ml		Y	Y	Y	Y	Y	
**Vc**	Sigma	49752	50 μg/ml		Y	Y	Y	Y	Y	
**βMe**	Gibco	21985-023	55 mM		Y	Y		Y	Y	
**cAMP**	Sigma	D-0260	1 μM							Y
**BDNF**	PeproTech	450-02	10 ng/ml							Y
**DAPT**	Selleck	S2215	5 μM							Y
**A8-301**	Sigma	SML0788	0.5 μM						Y	
**CHIR99021**	Selleck	S2924	3 μM						Y	
**VPA**	Calbiochem	676380	1 mM						Y	
**Forskolin**	Sigma	F3917	20 μM						Y Day 6	
**ITS**	Gibco	41400-045	I: 10 mg/L<br>T: 5.5 mg/L<br>S: 6.7 μg/L							
**Insulin**	Sigma	I8405	10 mg/L							
**Transferrin**	Sigma	T0665	5.5 mg/L							
**Na Selenite**	MPBIO	0219474110	6.7 μg/L							
**PD0332991**	Selleck	S1036								

Table S2

List of antibodies used in current study

Antibody Against	Company	Cat. No.	Application	Dilution	Ref.
Beta III Tubulin	Millipore	MAB5564	ICC, FACS	500X	[3]
Beta III Tubulin	Millipore	MAB1637	ICC	500X	[4]
GFAP	Millipore	MAB360	IHC	500X	[5]
GFAP	Millipore	04-1062	ICC	500X	[6]
GFAP	Dako	Z0334	IHC	500X	[7]
NeuN	Millipore	ABN78	ICC, IHC	500X	[8]
DCX	Cell Signaling	4604S	ICC	500X	[9]
GABA	Millipore	MAB316	ICC	500X	[10]
Glutamate	Millipore	AB1520	ICC	500X	[11]
Map2	Abcam	ab11267	ICC	500X	[12]
Nestin	Sigma	N5413	ICC	500X	[13]
NICD	Abcam	Ab52301	ICC	500X	[14]
O4	Millipore	MAB345	ICC	500X	[15]
Pax6	DSHB	PAX6	ICC	200X	[16]
Sox1	Millipore	AB15766	ICC	200X	[17]
Sox2	R&D	MAB2018	ICC	500X	[18]
Synapsin I	Millipore	AB1543	ICC	500X	[19]
Tyrosine Hydroxylase	Millipore	AB152	ICC	500X	[20]
Vim	Millipore	MAB1681	ICC	500X	[21]
Donkey@Rabbit Alexa 488	Invitrogen	A21206	IHC, ICC, FACS	400X	/
Donkey@Mouse Alexa 488	Invitrogen	A21202	IHC, ICC	400X	/
Donkey@Rabbit Alexa 568	Invitrogen	A10042	IHC, ICC	400X	/
Donkey@Mouse Alexa 568	Invitrogen	A10037	IHC, ICC	400X	/

Supplementary Reference

1. Wang L, Wang L, Huang W, Su H, Xue Y, Su Z *et al.*: Generation of integration-free neural progenitor cells from cells in human urine. ***Nature methods.*** 2013, **10**(1):84-89.
2. Liu X, Sun H, Qi J, Wang L, He S, Liu J *et al.*: Sequential introduction of reprogramming factors reveals a time-sensitive requirement for individual factors and a sequential EMT-MET mechanism for optimal reprogramming. ***Nat Cell Biol.*** 2013, **15**(7):829-838.
3. Silva J, Wang G, Cowell JK. The temporal and spatial expression pattern of the LGI1 epilepsy predisposition gene during mouse embryonic cranial development. ***BMC neuroscience.*** 2011, **12**(
4. Garcia I, Huang L, Ung K, Arenkiel BR. Tracing synaptic connectivity onto embryonic stem cell-derived neurons. ***Stem cells.*** 2012, **30**(10):2140-2151.
5. Karimi-Abdolrezaee S SD, Wang J, Fehlings MG.: Chondroitinase and growth factors enhance activation and oligodendrocyte differentiation of endogenous neural precursor cells after spinal cord injury. ***PLoS One.*** 2012, **7**(5):e37589.
6. Shi Y, Gelman BB, Lisinicchia JG, Tang SJ. Chronic-pain-associated astrocytic reaction in the spinal cord dorsal horn of human immunodeficiency virus-infected patients. ***The Journal of neuroscience : the official journal of the Society for Neuroscience.*** 2012, **32**(32):10833-10840.
7. Codeluppi S, Gregory EN, Kjell J, Wigerblad G, Olson L, Svensson CI. Influence of rat substrain and growth conditions on the characteristics of primary cultures of adult rat spinal cord astrocytes. ***Journal of neuroscience methods.*** 2011, **197**(1):118-127.
8. Najm FJ, Zaremba A, Caprariello AV, Nayak S, Freundt EC, Scacheri PC *et al.*: Rapid and robust generation of functional oligodendrocyte progenitor cells from epiblast stem cells. ***Nature methods.*** 2011, **8**(11):957-962.
9. Joseph G. Gleeson ea. <Doublecortin, a brain-specific gene mutated in human X-linked lissencephaly and double cortex syndrome, encodes a putative signaling protein.pdf>. ***Cell.*** 1998.
10. Westerholz S, de Lima AD, Voigt T. Regulation of early spontaneous network activity and GABAergic neurons development by thyroid hormone. ***Neuroscience.*** 2010, **168**(2):573-589.
11. Wernig M, Benninger F, Schmandt T, Rade M, Tucker KL, Bussow H *et al.*: Functional integration of embryonic stem cell-derived neurons in vivo. ***The Journal of neuroscience : the official journal of the Society for Neuroscience.*** 2004, **24**(22):5258-5268.
12. Regad T, Roth M, Bredenkamp N, Illing N, Papalopulu N. The neural progenitor-specifying activity of FoxG1 is antagonistically regulated by CKI and FGF. ***Nature cell biology.*** 2007, **9**(5):531-540.
13. Chen BY, Wang X, Wang ZY, Wang YZ, Chen LW, Luo ZJ. Brain-derived neurotrophic factor stimulates proliferation and differentiation of neural stem cells, possibly by triggering the Wnt/beta-catenin signaling pathway. ***Journal of neuroscience research.*** 2013, **91**(1):30-41.
14. Lasagni L, Ballerini L, Angelotti ML, Parente E, Sagrinati C, Mazzinghi B *et al.*: Notch activation differentially regulates renal progenitors proliferation and differentiation toward the podocyte lineage in glomerular disorders. ***Stem cells.*** 2010, **28**(9):1674-1685.
15. Giannotta M, Benedetti S, Tedesco FS, Corada M, Trani M, D'Antuono R *et al.*: Targeting endothelial junctional adhesion molecule-A/ EPAC/ Rap-1 axis as a novel strategy to increase stem cell engraftment in dystrophic muscles. ***EMBO molecular medicine.*** 2014, **6**(2):239-258.
16. Englund C, Fink A, Lau C, Pham D, Daza RA, Bulfone A *et al.*: Pax6, Tbr2, and Tbr1 are expressed sequentially by radial glia, intermediate progenitor cells, and postmitotic neurons in developing neocortex. ***The Journal of neuroscience : the official journal of the Society for Neuroscience.*** 2005, **25**(1):247-251.
17. Liu J, Koscielska KA, Cao Z, Hulsizer S, Grace N, Mitchell G *et al.*: Signaling defects in iPSC-derived fragile X premutation neurons. ***Human molecular genetics.*** 2012, **21**(17):3795-3805.
18. Suh H, Consiglio A, Ray J, Sawai T, D'Amour KA, Gage FH. In vivo fate analysis reveals the multipotent and self-renewal capacities of Sox2+ neural stem cells in the adult hippocampus. ***Cell stem cell.*** 2007, **1**(5):515-528.
19. Xue Y, Ouyang K, Huang J, Zhou Y, Ouyang H, Li H *et al.*: Direct conversion of fibroblasts to neurons by reprogramming PTB-regulated microRNA circuits. ***Cell.*** 2013, **152**(1-2):82-96.

20. Wen Z, Nguyen HN, Guo Z, Lalli MA, Wang X, Su Y *et al.*: Synaptic dysregulation in a human iPS cell model of mental disorders. ***Nature***. 2014, **515**(7527):414-418.
21. K. ASAUMI TN, H. ASAHARA, H. INOUE,and M. TAKIGAWA. Expression of Neurotrophins and Their Receptors (TRK) During Fracture Healing. ***MOLECULAR CELL***. 2000.
